# Tuberculosis parenteral therapeutic regimens for critical patients or non-functional intestinal tract: Brief review and proposal of protocol

**DOI:** 10.1016/j.bjid.2025.104526

**Published:** 2025-04-07

**Authors:** Antonio Camargo Martins, Márcia Teixeira Garcia, Mariângela Ribeiro Resende

**Affiliations:** a Ambulatório de Referência Regional de TB-MDR/MNT do Hospital de Clínicas da Universidade Estadual de Campinas (UNICAMP), Campinas, SP, Brasil; b Infectologia, Departamento de Clínica Médica, Faculdade de Ciências Médicas (FCM), Universidade Estadual de Campinas (UNICAMP), Campinas, SP, Brasil; c Rede Brasileira de Pesquisa em Tuberculose (REDE-TB), Rio de Janeiro, RJ, Brasil; d Programa de Tuberculose de Indaiatuba, Secretaria de Saúde de Indaiatuba, Indaiatuba, SP, Brasil; e Saúde Coletiva, Faculdade de Medicina, Centro Universitário Max Planck (UNIMAX), Indaiatuba, SP, Brasil; f Infectologia, Hospital Municipal Dr Mário Gatti, Campinas, SP, Brasil; g Infectologia, Faculdade de Medicina, Faculdade São Leopoldo Mandic, Campinas, SP, Brasil

**Keywords:** Tuberculosis, Intensive care unit, Intravenous, Treatment, Non-functional intestinal tract

## Abstract

Standard anti-tuberculosis regimens (Rifampicin, Isoniazid, Pyrazinamide, and Ethambutol [RHZE]) remain challenging for critically ill patients and those with a non-functioning gastrointestinal tract. In Brazil, these challenges are amplified by the lack of Intravenous (IV) rifampicin, isoniazid, and ethambutol, which often results in suboptimal outcomes. This brief communication synthesized evidence on parenteral therapies and proposed a structured, five-step protocol for critically ill patients unable to receive oral drugs. A narrative review of the guidelines and key studies was also conducted. IV formulations of RHZE are approved in only some countries and are not available everywhere. Alternative IV drug classes, such as fluoroquinolones, aminoglycosides, carbapenems, and oxazolidinones, can address malabsorption or intolerance to oral RHZE. However, no standardized regimen exists for this population. Our five-step protocol advises: (1) Characterizing each TB case, (2) determining IV necessity, (3) Consulting specialized TB services, (4) Designing a safe and effective regimen, and (5) *Re*-evaluating therapy for transition to oral treatment. Given the morbidity and mortality from severe TB in Intensive Care Units (ICU), a formalized approach is essential. Further research and policy initiatives regarding IV first-line drugs are crucial to improve treatment outcomes in this vulnerable group. This strategy unifies practice across diverse clinical settings.

Global goals and expectations for tuberculosis (TB) control, including improvements in diagnosis, reductions in transmission, and decreases in mortality, have been significantly set back due to the impact of the coronavirus disease 2019 pandemic.^1^ A similar trend has been observed in Brazil, which has had the highest increase in mortality rates over the past decade.^2^ Late diagnosis remains a persistent issue in various healthcare services, leading to the advancement of TB stages^3^, unfavorable prognosis, and ultimately, mortality.^1^ Consequently, there has been an increase in severe cases of disseminated, meningeal, and intestinal TB, leading to the need for hospital care, often in ICUs. From 2018 to 2023, at the Hospital de Clínicas da Universidade Estadual de Campinas (HC-UNICAMP), 68 patients were admitted to the ICU (unpublished data), necessitating the formulation of decisions regarding the most appropriate regimen for these cases.

This report aimed to provide a concise and narrative review of the therapeutic possibilities for TB in ICU patients who lack a functioning gastrointestinal tract. This includes individuals with critical conditions such as shock, abdominal surgery, and chronic intestinal diseases, whether associated with or independent of TB, who cannot absorb oral medications. Various national and international protocols for TB treatment were reviewed to propose a therapeutic management protocol for these situations.

The mortality rate of TB patients in ICUs is typically > 50 %.^4^, ^5^, ^6^, ^7^ Two therapeutic difficulties are associated with this group of patients: the first is related to erratic absorption of drugs due to shock or organ dysfunction, and the second involves making necessary adjustments for renal or hepatic impairment.^4^^,^^8^^,^^9^ Additionally, more severely ill patients (APACHE II score > 18) are approximately twice as likely to have Rifampicin, Isoniazid, Pyrazinamide, and Ethambutol (RHZE) regimen suspended because of the adverse effects of the medication.^10^

A common challenge in critically ill patients is the need to administer macerated medications via the Nasogastric Tube (NGT). This process, particularly in the context of the RHZE scheme, can potentially lead to reduced drug absorption. The medication most affected in this situation is rifampicin^11^, which when administered via the NGT with food, does not reach therapeutically effective levels.^12^ Even when administered on an empty stomach (as recommended), only 20 % of patients reach the expected therapeutic dose.^12^ Other compromised medications include ethambutol and isoniazid.^11^ In the case of gastrointestinal TB, the standard6-month RHZE regimen has been validated as effective^13^; however, in some situations, the severity of gastrointestinal TB involvement can lead to malabsorption, necessitating the use of Intravenous (IV) medications.^14^

In summary, the management of ICU patients with TB is inherently complex and hampered by concerns such as drug toxicity, erratic absorption, organ dysfunction, and limited capacity for therapeutic-level monitoring.^9^ A systematic review by Galvin et al. (2022) suggested that careful use of IV medications could help reduce complications and mortality in these cases, potentially offering an alternative approach for high-risk populations.^4^

In 2023, the World Health Organization (WHO) emphasized the importance of the availability of IV drugs for severe cases, such as in situations where absorption is difficult.^15^ However, it is important to note that the three medications currently available for IV therapy (R, H, and E) have not been approved for inclusion in the WHO Model List of Essential Medicines (23rd List, 2023). Although IV formulations are particularly important in specific cases, this group of patients does not represent the majority of TB cases, thus providing a rationale for their exclusion.^16^, ^17^, ^18^, ^19^

IV presentations are available in different countries and are regulated by specific agencies. These include R in the U.S., regulated by the Food and Drug Administration (FDA)^20^; H in the U.S. and some European countries, regulated by the FDA and European Medicines Agency (EMA), respectively^21^; and E in parts of Europe, regulated by the EMA.^22^ In Brazil, these drugs still need to be made available and approved by the National Health Surveillance Agency.

The fact that most TB patients can tolerate oral formulations, it is not surprising that the current drug development pipelines emphasize this route of administration.^23^^,^^24^ For instance sudapyridine, an oral agent, has progressed to phase III trials and is actively recruiting patients.^25^ However, promising IV alternatives exist among the novel oxazolidinone classes, notably, tedizolid and delpazolid.^24^^,^^26^ Although both compounds have documented IV formulations, ongoing clinical studies are primarily testing them via oral routes; tedizolid is in phase II trials^27^, while delpazolid has recently completed this phase.^28^ Consequently, limited data are available on the parenteral forms, leaving a significant gap for critically ill populations that may require IV therapy.

Although the current TB treatment guidelines lack comprehensive recommendations for IV anti-TB drugs for critically ill patients^8^^,^^29^, ^30^, ^31^, ^32^, several studies have demonstrated that such agents are used in practice. In Brazil, for example, two studies documented IV therapy in ICU settings; one reported that 63 % of HIV-positive patients received aminoglycosides or fluoroquinolones^6^, whereas a retrospective case series reported IV regimens in 38.5 % of cases.^5^ In the United Kingdom, Hagan and Nathani observed that IV therapy was initiated within the first 72 h of ICU admission^9^, and a Chinese case series noted that 10 % of patients received Levofloxacin (Lfx) or streptomycin.^7^ Despite the frequent use of parenteral drugs among severely ill TB patients, no standardized protocol has been established in clinical trials, and large-scale data remain limited.

Many countries, including Brazil, lack access to essential IV formulations, further complicating the treatment of critically ill patients. Recognizing this gap, we proposed a therapeutic strategy for patients requiring IV interventions. Notably, this protocol, which was designed for a specific subgroup, has not yet been formally validated.

The following five sequential steps were proposed to systematically and individually determine the indications for IV therapy: characterization of TB cases, determination of the necessity of IV therapy, TB reference technical assistance, design of the regimen and therapeutic proposal, and re-evaluation of treatment once clinical improvement has occurred (Fig. 1).

**Fig. 1:**
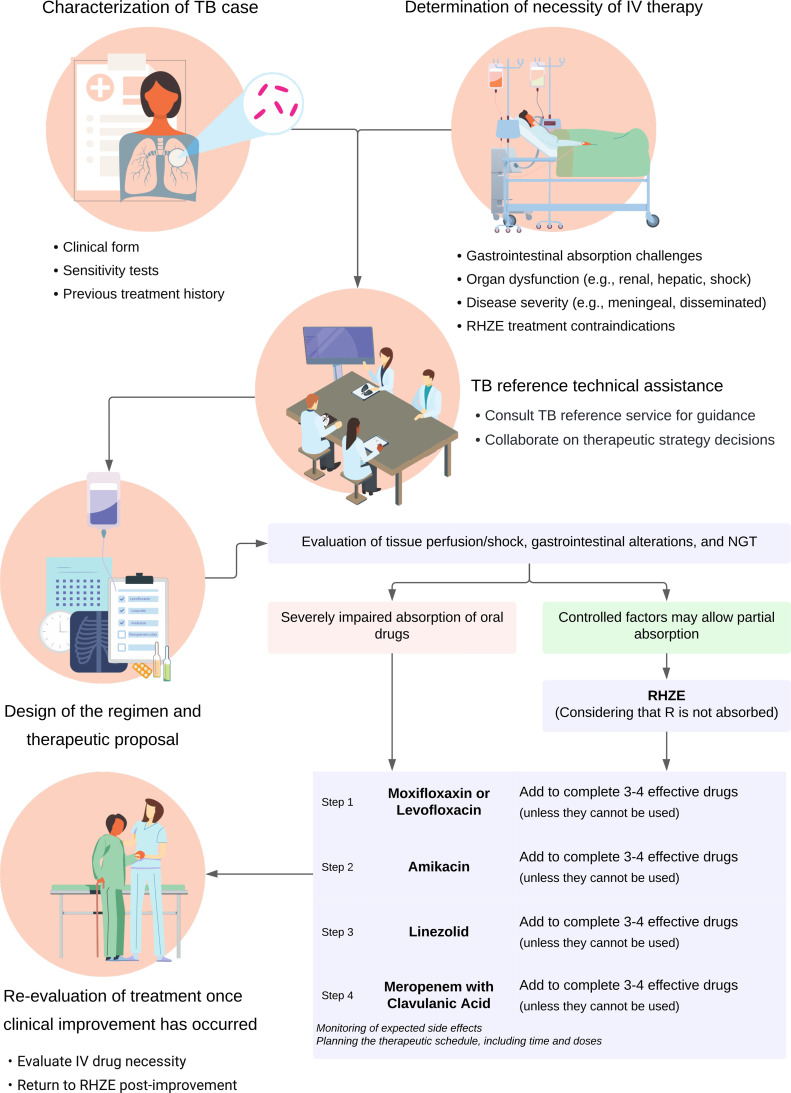
Five steps to systematically assess intravenous therapy indications.

## Characterization of TB case

The initial step is to individualize the case of TB, which comprises an assessment of a range of variables. These include the clinical form of TB, its severity, the patient's treatment history (cure or previous abandonment), the results of diagnostic tests, and the drug sensitivity profile. At this juncture, patient variables will also be characterized, including comorbidities, HIV status, and use of medications that may interact with anti-TB drugs. Specific protocols should be employed in cases of confirmed resistance.

## Determination of necessity of IV therapy

Determining the necessity of IV therapy is the most complex step in this process, as no clinical trials have defined which patients benefit from the use of IV drugs. Therefore, decisions regarding the use of IV drugs in different services are made according to expert opinions. The serum levels of anti-TB drugs in critically ill patients are not known; however, it is prudent to consider that, due to various organ dysfunctions, drugs administered orally must be at sub-therapeutic levels.^9^

It is recommended that patients who require intensive care be considered for the individualization of therapeutic regimens, particularly those with organic dysfunctions (gastrointestinal, hepatic, renal, and circulatory) that could result in malabsorption, or where medication presents a risk of impairment of these dysfunctions. Furthermore, the negative effects of NGT use on absorption should also be considered.^11^^,^^12^

## TB reference technical assistance

The third and fourth phases should be performed simultaneously as the therapeutic program continues. Therefore, therapeutic planning should be discussed with the TB referral service. This recommendation is essential, given the complexities of designing a safe and effective regimen that minimizes the risk of developing resistance. This recommendation is made by the Official American Thoracic Society/Centers for Disease Control and Prevention/Infectious Diseases Society of America Clinical Practice, which advises discussions with referral centers in complex diagnostic and management situations.^32^

## Design of the regimen and therapeutic proposal

The selection of drugs for TB treatment regimens should follow specific principles. Initially, there should be three to four effective drugs that not only eliminate *Mycobacterium tuberculosis* but also prevent the development of resistance.^31^ Drugs should be selected based on their bactericidal activity to rapidly reduce the bacillary load, reduce disease severity, prevent fatal complications, and improve symptoms.^32^ In addition to drug efficacy, it is essential to consider the possible adverse events and interactions with other drugs.

In Brazil, only four classes of IV drugs are available for the treatment of TB: fluoroquinolones, aminoglycosides, carbapenems, and oxazolidinones. These drugs are commonly used to treat bacterial infections in hospitals. However, it is pertinent to note that the recommended therapeutic doses for TB may differ from those recommended for other infections (Table 1). In cases of meningoencephalitic TB, adjustments may be necessary to ensure optimal penetration into the CNS.

**Table 1:** Intravenous drugs for tuberculosis treatment.

* Adapted from Caminero et al., Arch Bronconeumol, 2020^31^ and WHO^29^.

In formulating an individualized regimen, fluoroquinolones (Moxifloxacin [Mfx], and Levofloxacin [Lfx]) should be considered as the primary option, given their demonstrated efficacy in terms of bactericidal activity and low toxicity, as well as their minimal propensity to interact with other drugs. In terms of therapeutic success, the two proposed quinolones are very similar^33^, ^34^, ^35^, with subtle differences. Mfx is associated with faster conversion to negative cultures^33^, ^36^ and lower mortality^34^; however, it is most commonly associated with arrhythmias when used alone.^37^ In contrast, Lfx has fewer adverse effects.^33^ Ciprofloxacin is not recommended for the treatment of *M. tuberculosis* because of its low intracellular activity^38^ and the rapid emergence of resistance.^39^ Additionally, Mfx and Lfx represent viable alternatives for transitioning to oral administration regimens, particularly when their use is indicated owing to adverse events associated with other drugs.

Amikacin (Am) exhibits potent bactericidal activity by irreversibly binding to the 30S ribosomal subunit of *M. tuberculosis*, resulting in tRNA misreading and disruption of protein synthesis.^29^^,^^40^ However, its use is associated with considerable nephrotoxicity and ototoxicity, particularly in individuals aged > 60-years. Consequently, close monitoring is essential to mitigate the risk of irreversible adverse effects. Current recommendations advise administering Am no more than three times per week, adjusting doses for older adults, and limiting treatment duration to a brief interval.^29^^,^^41^

Linezolid (Lzd), an oxazolidinone traditionally used to treat *Staphylococcus aureus* infections, has demonstrated notable efficacy against *M. tuberculosis*. It inhibits bacterial protein synthesis by binding to the 23S ribosomal RNA of the 50S ribosomal subunit, blocking mRNA reading, and initiating protein production.^29^^,^^40^ It has an intermediate toxicity profile with adverse events including myelotoxicity and thrombocytopenia. In the context of TB, clinical trials have indicated that 600 mg/day of Lzd is both efficacious and less toxic, establishing this dosage as the recommended dosage.^29^^,^^42^^,^^43^ Similar to fluoroquinolones, Lzd is available in both IV and oral formulations and has good bioavailability, making it a viable alternative for transitioning from IV to oral regimens.

Carbapenems, notably, Meropenem (Mpm) and Imipenem (Imp), exhibit moderate bactericidal activity compared with other pharmaceutical agents. The efficacy of carbapenems is contingent upon their use in conjunction with clavulanic acid due to the production of β-lactamase BlaC by *M. tuberculosis*. Carbapenem toxicity is generally low, although Imp use is associated with a decreased seizure threshold, particularly in patients with epilepsy. Mpm is more effective than Imp in regimens and should therefore be the preferred option.^44^ Carbapenems should be added as a last choice to IV regimens, as they have less potent action, and there are additional implications related to the selection of Multidrug-Resistant microorganisms in intensive care.

A combined regimen of RHZE and IV drugs can be considered, provided that critical factors such as tissue perfusion or shock, gastrointestinal structural and metabolic changes, and the use of the NGT, are carefully evaluated.^45^ For patients whose hemodynamic and gastrointestinal conditions have stabilized, oral administration, either conventionally or via the NGT, is generally recommended. Each RHZE component remains effective, except for R, which tends to show reduced absorption through the NGT. When IV therapy is indicated, the previously proposed protocol sequence should be followed. Additionally, these medications should ideally be administered on an empty stomach, 2‒3 h before and after meals to enhance absorption.^12^^,^^46^^,^^47^

In summary, the optimal therapeutic regimen should comprise three to four efficacious drugs selected based on their superior bactericidal activity and reduced toxicity. In a scenario where RHZE is not viable, the recommended parenteral regimen comprises a fluoroquinolone, Am, and/or Lzd, and/or carbapenem with clavulanate. However, when RHZE is viable, its administration via the NGT can be complemented by IV drugs according to the outlined order. It is important to evaluate the potential adverse effects of these drugs, which may exacerbate pre-existing conditions (hepatotoxicity of RHZ, nephrotoxicity of Am, and myelotoxicity of Lzd), along with possible drug interactions^48^, ^49^, ^50^, particularly in patients receiving intensive care (Table 2).

**Table 2:** Interactions of intravenous drugs used in tuberculosis treatment.

## Pediatric population

In pediatric populations, as in adults, there is a notable scarcity of data on IV TB treatment. Existing recommendations largely rely on guidelines designed for drug-resistant TB, and most clinical experience with these interventions pertains to children older than 5-years.^51^^,^^52^ Paradoxically, the highest mortality rates occur in children aged 0 to 4 years.^53^ The WHO supports the use of Mfx, Lfx, Am, Lnz, Mpm, and Imp in pediatric and adolescent cases of resistant TB.^54^^,^^55^ Nevertheless, concerns regarding ototoxicity have limited the recommended use of Am in individuals aged > 18 years.^54^ Additionally, there is a paucity of data on the risk of fluoroquinolone-induced arthropathy in children aged < 5-years.^52^

## MDR-TB

In MDR-TB settings, the IV drugs outlined in this protocol are commonly used in oral formulations, often in combination with one or more medications, such as bedaquiline^56^, ^57^, ^58^, delamanid^59^^,^^60^, pretomanid^61^, and terizidone^62^, that can be diluted for administration. The current dilution guidelines provide limited information on the feasibility of NGT delivery. Nonetheless, for critically ill MDR-TB patients in intensive care, these agents may be administered through the NGT to augment the therapeutic regimen, although their bioavailability via this route remains uncertain owing to the lack of specific studies.

## *Re*-evaluation of the treatment once clinical improvement has occurred

The precise duration of IV treatment has not been established; therefore, it is recommended that it be used for the shortest feasible period and that the transition to oral treatment with RHZE be initiated as soon as possible. Following recovery, a thorough review should determine whether maintenance therapy is necessary and whether any adjunctive medications can be discontinued or continued. Standard RHZE alone is generally advised in patients who have recovered from critical illness and no longer face malabsorption issues.

## Final considerations

In the absence of robust trials defining optimal IV regimens for critically ill patients with TB, our proposed protocol highlights the need for individualized strategies. Although the data remain limited, it is evident that severe cases frequently require alternative routes of administration.

Although this protocol has not yet been validated in large-scale clinical trials, it underscores the potential cost-effectiveness of IV R and H for patients with critical illnesses, especially in settings where regulatory constraints limit access to first-line IV drugs. Alternative parenteral agents are indispensable when oral administration is infeasible. Ultimately, we aim to advance the development of safer and more effective treatment regimens for high-risk populations, calling attention to the urgent need for the broader availability of IV TB medications in regions where they remain inaccessible.

## Conflicts of interest

The authors declare no conflicts of interest.
